# Overt diabetes imposes a comparable burden on outcomes as pregestational diabetes: a cohort study

**DOI:** 10.1186/s13098-022-00939-1

**Published:** 2022-11-23

**Authors:** Maria Lúcia Oppermann, Maria Amélia Campos, Vânia Naomi Hirakata, Angela Jacob Reichelt

**Affiliations:** 1 Serviço de Ginecologia e Obstetrícia, Hospital de Clínicas de Porto Alegre, Ramiro Barcelos, 2350, Porto Alegre, 90035-903 Brazil; 2 Faculdade de Medicina, Universidade Federal do Rio Grande do Sul, Rua Ramiro Barcelos, 2400, Porto Alegre, 90035-003 Brazil; 3 Serviço de Endocrinologia e Metabologia, Hospital Nossa Senhora da Conceição, Av. Francisco Trein, 596, Porto Alegre, 91350-200 Brazil; 4 Unidade de Bioestatística, Hospital de Clínicas de Porto Alegre, Ramiro Barcelos, 2350, Porto Alegre, 90035-903 Brazil; 5 Serviço de Endocrinologia e Metabologia, Hospital de Clínicas de Porto Alegre, Centro de Pesquisa Experimental, Ramiro Barcelos, 2350, Prédio 12, 4º. Andar, Porto Alegre, CEP 90035-903 Brazil; 6 Programa de Pós-Graduação em Ciências Médicas: Endocrinologia, Universidade Federal do Rio Grande do Sul, Porto Alegre, Brazil

**Keywords:** Pregestational diabetes, Overt diabetes, Pregnancy outcomes

## Abstract

**Background:**

Women with diabetes first diagnosed during pregnancy (overt diabetes) may be at the same risk level of adverse outcomes as those with known pregestational diabetes. We compared pregnancy outcomes between these groups.

**Methods:**

We evaluated pregnant women with type 2 diabetes, pregestational or overt diabetes, attending high risk antenatal care in two public hospitals in Southern Brazil, from May 20, 2005 to June 30, 2021. Outcomes were retrieved from electronic medical records. Risk of adverse outcomes, expressed as relative risk (RR) and 95% confidence interval (CI), were calculated using Poisson regression with robust estimates.

**Results:**

Of 618 women, 33% were labelled as having overt diabetes and 67%, pregestational diabetes. Baseline maternal characteristics were similar: there was a slight, non-clinically relevant, difference in maternal age (33 ± 5.7 years in women with pregestational diabetes vs. 32 ± 6.0 years in women with overt diabetes, p = 0.004); and women with overt diabetes reported smoking almost twice compared to those with pregestational diabetes (12.3% vs. 6.5%, p = 0.024). There were no relevant differences between the groups regarding pregnancy outcomes, although there was a trend of higher neonatal intensive care admission in the group of women with pregestational diabetes (45.2% vs. 36.1%, p = 0.051).

**Conclusions:**

Overt diabetes was diagnosed in one third of this cohort of pregnant women with hyperglycemia. Their pregnancy outcomes were similar to those of women with pregestational diabetes and were mostly related to maternal demographic characteristics and metabolic control. A call to action should be made to identify women of childbearing age at risk for pre-pregnancy diabetes; to detect hyperglycemia before conception; and to implement timely preconception care to all women with diabetes.

**Supplementary Information:**

The online version contains supplementary material available at 10.1186/s13098-022-00939-1.

## Background

Diabetes associated to pregnancy may carry adverse maternal and neonatal outcomes. Some of them, such as preeclampsia, preterm delivery, large babies, and perinatal mortality are at least three times more frequent in women with pregestational diabetes than in those without diabetes [1].

Type 2 diabetes, in an overall upwards trend paralleling that of obesity, now affects many women of childbearing age [2]. All women with overweight or obesity should, ideally, be screened for undiagnosed hyperglycemia before conception, or at least, in early pregnancy [2, 3]. In pregnancy, criteria for diabetes diagnosis are the same as for non-pregnant subjects; this situation is labelled as “overt diabetes” [4] or as “diabetes in pregnancy” [5] or as “diabetes complicating pregnancy” [3].

Women with overt diabetes may be at the same risk of adverse outcomes as those with known pregestational diabetes [6]; despite this, they have been excluded from studies of pregnancies in women with type 2 diabetes [7, 8].

We aimed to compare main pregnancy outcomes of women with overt diabetes to those of women with known pregestational type 2 diabetes.

## Methods

We retrospectively evaluated consecutive pregnant women with type 2 diabetes attending high risk antenatal care in the two major public hospitals (Hospital de Clínicas de Porto Alegre (HCPA) [9] and Hospital Nossa Senhora da Conceição (HNSC) [10]) of Porto Alegre, Brazil, from May 20, 2005 to June 30, 2021.

The ethics committee of both hospitals approved the study protocol on July 28, 2016 (number 16-0331) and the study is registered at Plataforma Brasil, CAAE 57365016.3.0000.5327; all authors signed a data use agreement form to ensure privacy of data collected from medical registries.

All women with the typical clinical features and a pregestational diagnosis of type 2 diabetes were enrolled [11]; and all those fulfilling the 2013 World Health Organization criteria for overt diabetes (fasting plasma ≥ 126 mg/dl or 2 h glucose after a 75 g load ≥ 200 mg/dl) [5] and/or the American Diabetes Association recommendation of glycated hemoglobin (HbA1c) ≥ 6.5% [3]. We could not detect maturity-onset diabetes of the youth (MODY) due to technical limitations; therefore, we used clinical characteristics attributable to type 2 diabetes as a proxy for diagnosis to include those women. We excluded women with type 1 diabetes diagnosis and those with clinical and/or laboratorial features of latent autoimmune diabetes of adulthood (LADA); and those with gestational diabetes or an inaccurate diagnosis of hyperglycemia. If a woman became pregnant more than once during the study span, we only included data of the first pregnancy. In both hospitals, a multi-professional team provided antenatal care.

Data were retrieved from electronic medical records. Duration of diabetes and pre-pregnancy weight were informed at the first prenatal appointment. Presence of any diabetes complications, smoking, family history of diabetes or chronic hypertension, personal history of hypertension, previous gestational diabetes or macrosomia (birth weight ≥ 4000 g) were considered positive when recorded in the hospital chart. The same was applied for family history of diabetes or hypertension in relatives of first or second degree. The absence of information on these variables was labeled as negative.

Height was measured at the first prenatal appointment, and weight, at each visit. Pregestational body mass index (BMI) was calculated as the informed pregestational weight in kilograms divided by the square of height, in meters, and women were classified as having normal BMI, overweight, or obesity [12]. Gestational weight gain adequacy was classified according to the 2009 National Academy of Medicine recommendation [12].

HbA1c was measured at booking, regardless of gestational age; and repeated at least once more beyond the 28th week. Assays were conducted with high-performance liquid chromatography (Variant II Turbo HbA1c; BioRad, Hercules, CA, USA) in line with the National Glycohemoglobin Standardization Program guidelines (http://www.ngsp.org/index.asp).

All pregnancy outcomes were retrieved from the hospitals’ electronic records; we assumed the diagnosis as recorded by the medical teams. Birth weight categories were classified according to the World Health Organization chart [13], and congenital malformations, by the 10^th^ revised International Classification of Diseases, Q chapter. Perinatal and neonatal death were labelled as death, preterm birth as delivery with less than 37 gestational weeks and macrosomia as birthweight ≥ 4000 g.

The manuscript was written following the Strengthening the Reporting of Observational Studies in Epidemiology (STROBE) guideline [14].

### Statistical analysis

We compared pregnancy evolution and maternal and fetal/perinatal outcomes of women with overt diabetes to those with known pregestational type 2 diabetes in univariable analysis; and we assessed risks of adverse outcomes using Poisson regression with robust estimates, setting overt diabetes as the dependent variable. Women with unknown time of diabetes, with multiple pregnancies and those with miscarriage were excluded from all analysis of outcomes.

Statistical analyses were performed with SPSS version 18.0 (SPSS, Chicago, IL, USA). Data were expressed as mean ± standard deviation (SD) or median [interquartile range, IQR] according to normal distribution as determined by Shapiro–Wilk test, or number (percentage). The Student *t* test, the chi-square test (coupled with *Z* test for comparison of proportions, with Bonferroni correction when appropriate), and the Mann–Whitney *U* test were used to compare outcomes of women with overt diabetes to those with pregestational diabetes and for the comparisons of the HbA1c; multivariable analyses are expressed as relative risk (RR) and 95% confidence interval (CI).

## Results

We enrolled 648 women; we excluded two women with unknown time of diabetes diagnosis, those with pregnancies resulting in miscarriage (n = 18) and those with multiple pregnancies (n = 10), leaving 618 (95.4%, 95% CI 93.5–96.9%) women. Of the 618 women, 284 (46%) were from HCPA and 334 (54%), from HNSC; 204 (33%) women were diagnosed with overt diabetes and 414 (67%), with pregestational diabetes. Overt diabetes was diagnosed at a median gestational age of 12.3 weeks [IQR 8.3–19.0]; most women were diagnosed in the first trimester (n = 111, 54.4%) and 30 (14.7%), at or after 24 weeks. The combination of high fasting plasma glucose (FPG) + HbA1c was the most frequent diagnostic criterion (69 women, 33.8%); others included only FPG, 33 women (16.2%); 2 h in the oral glucose tolerance test (OGTT), 28 (13.7%); only HbA1c, 18 women (8.8%); FPG + 2 h OGTT, 13 women (6.4%); 2 h and HbA1c, 7 women (3.4%); and all criteria, 36 women (17.6%).

Baseline characteristics of the groups are presented in Table 1. There was a slight, non-clinically relevant, difference in maternal age (32 ± 6.0 years in women with overt diabetes vs. 33 ± 5.7 years in women with pregestational diabetes, p = 0.004). Women with overt diabetes reported smoking almost twice compared to those with pregestational diabetes (12.3% vs. 6.5%, p = 0.024). Information regarding treatment was available for 409 (98.7%) of the 414 women with pregestational diabetes: 60.6% reported using oral medications; 10.0%, insulin; 15.4%, both treatments; 5.6%, diet only; and 8.3%, no treatment.

**Table 1:** Baseline characteristics of pregnant women with diabetes according to time of diagnosis

Characteristic		Diabetes		p
	All n = 618 (100)	Pregestational n = 414 (67)	Overt n = 204 (33)	
Center of enrollment				0.700
HCPA	284 (46.0)	193 (68.0)	91 (32)	
HNSC	334 (54.0)	221 (66.2)	113 (33.8)	
Age	33 (5.9)	33 (5.7)	32 (6.0)	0.004
White skin color	433(70.1)	288 (69.6)	145 (71.1	0.770
Schooling (≤ 11 years)	588 (95.1)	391 (94.4)	197 (96.6)	0.339
Smoking	52 (8.4)	27 (6.5)	25 (12.3)	0.024
Length of diagnosis (years)		4.0 [2.0–7.0]	–	–
		413		
Diabetes complications				0.003
none	581 (94.0)	379 (91.5)	202 (99.0)	
retinopathy	24 (3.9)	23 (5.6)	1 (0.5)	
nephropathy	10 (1.6)	9 (2.2)	1 (0.5)	
retinopathy + nephropathy	3 (0.5)	3 (0.7)	0 (0.0)	
Family history of diabetes	416 (67.3)	291 (70.3)	125 (61.3)	0.031
Family history of CH	309 (50.0)	213 (51.4)	96 (47.1)	0.347
Number of pregnancies	3.1 (1.7)	3.1 (1.8)	3.0 (1.6)	0.205
Previous miscarriage	174 (28.2)	121 (29.2)	53 (26.0)	0.454
Previous macrosomia	124 (20.1)	75 (18.1)	49 (24.0)	0.106
Chronic hypertension	143 (23.1)	108 (26.1)	35 (17.2)	0.018
Previous gestational diabetes	195 (31.6)	133 (32.1)	62 (30.4)	0.731
First pregnancy	114 (18.4)	76 (18.4)	38 (18.6)	> 0.999
Pregestational BMI	34.3 (7.7)	34.2 (7.4)	34.7 (8.2)	0.396
	596	399	197	
BMI categories				0.718
normal	55 (9.2)	36 (9.0)	19 (9.6)	
overweight	117 (19.6)	82 (20.6)	35 (17.8)	
obesity	424 (71.1)	281 (70.4)	143 (72.6)	
	596	399	197	
Gestational age at booking	19.6 [14.0–27.4]	18.0 [12.9–24.0]	25.0 [18.0–31.2]	< 0.001
Trimester of booking				< 0.001
first	123 (19.9)	103 (24.9)	20 (9.8)	
second	275 (44.5)	198 (47.8)	77 (37.7)	
third	220 (35.6)	113 (27.3)	107 (52.5)	
Initial HbA1c	7.2 (1.5)	7.4 (1.7)	7.0 (1.3)	0.008
	612 (99.0)	409	203	
Weight gain (booking)	2.9 [0.1–6.0]	2.6 [0.0–5.2]	4.0 [0.2–8.4]	0.003
	597	400	197	

Results presented as mean (standard deviation), n (%) or median (interquartile range)

*HCPA* Hospital de Clínicas de Porto Alegre, *HNSC* Hospital Nossa Senhora da Conceição, *CH* chronic hypertension, *BMI* body mass index, *HbA1c* glycated hemoglobin

Statistics: Student *t* test, the chi-square test (coupled with *Z* test for comparison of proportions, with Bonferroni correction when appropriate), and the Mann–Whitney *U* test

**Fig. 1:**
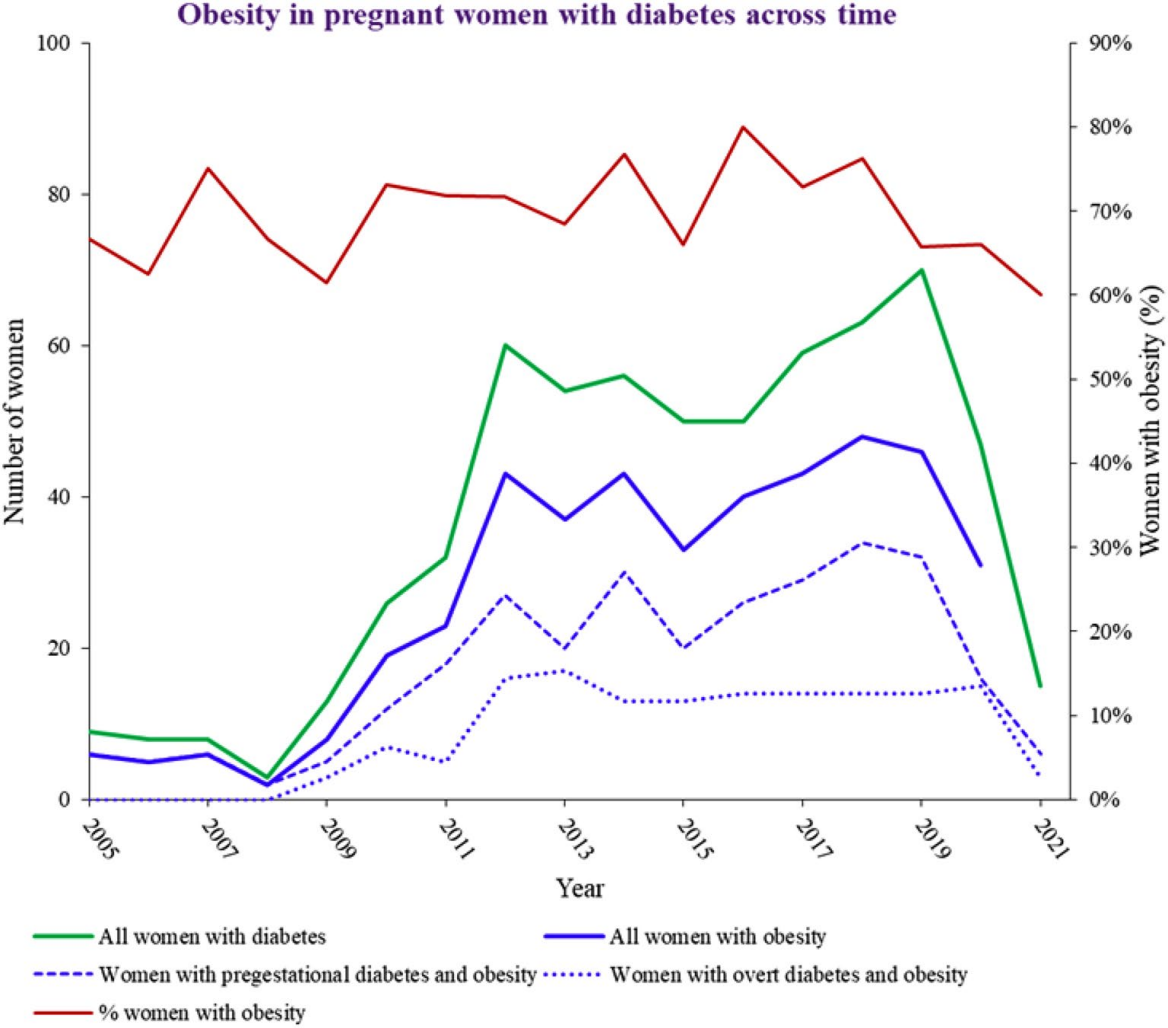
Obesity in pregnant women with overt and pregestational diabetes across time in two prenatal facilities in Porto Alegre, Brazil

Most women with overt diabetes (52.5%) arrived to the specialized prenatal care in the third trimester; 24.9% of women with pregestational diabetes arrived in the first trimester. The rate of obesity was high (424/596 women, 71.1%, 95% CI 67.0–75.0%), varied between 60.0 and 80.0% per year, but rates were similar across time (p = 0.967) (Fig. 1).

HbA1c at booking was available for 612 of the 618 women; 5 (0.8%) had preconception values; 207 (33.8%) were measured before 13 gestational weeks, 246 (40.2%), up to 24 gestational weeks and 154 (25.2%) at gestational age ≥ 24 weeks. HbA1c at booking was ≥ 6.5% in 402 of 612 women (65.7%).

Regarding characteristics according to center of enrollment (Additional file 1: Table S1), there were some differences in baseline maternal characteristics; however, except for preeclampsia rates (HCPA n = 69 (25.7%) vs. HNSC n = 128 (39.3%), p = 0.001) and insulin use at delivery (HCPA n = 244 (88.1%) vs. HNSC 267 (81.2%), p = 0.026), all other maternal and neonatal outcomes were similar.

Pregnancy follow-up and main maternal and neonatal outcomes are shown in Table 2. Pregnancy evolution and outcomes were similar between the two groups; 562 (90.9%) women delivered liveborn and 17 (2.8%), stillborn infants; 39 mother-baby pairs (6.3%) were lost to follow-up. At least one adverse outcome was noticed in 41.9% of the neonates.

**Table 2:** Pregnancy follow-up and outcomes in women with diabetes according to time of diagnosis

Outcome		Diabetes		p
	All	Pregestational	Overt	
	n = 618 (100)	n = 414 (67)	n = 204 (33)	
Center of enrollment				0.700
HCPA	284 (46.0)	193 (46.6)	91 (44.6)	
HNSC	334 (54.0)	221 (53.4)	113 (55.4)	
**Maternal outcomes**				
Specialized appointments	7.0 [3.0–10.0]	8.0 [4.0–11.0]	5.0 [2.0–9.0]	< 0.001
	617	413	204	
Insulin use	511 (84.3)	347 (85.3)	164 (82.4)	0.432
	606	407	199	
Hospitalization due to diabetes	344 (58.7)	234 (59.2)	110 (57.3)	0.771
	586 (94.8)	395	191	
Total weight gain	8.2 (7.5)	8.2 (6.7)	8.1 (9.0)	0.929
	565	378	187	
Weight gain categories^a^				0.145
less than recommended	211 (37.3)	131 (34.7)	80 (42.8)	
as recommended	147 (26.0)	100 (26.5)	47 (25.1)	
more than recommended	207 (36.6)	147 (38.9)	60 (32.1)	
	565	378	187	
HbA1c (≥ 28 weeks)	6.3 (0.9)	6.3 (0.9)	6.3 (1.0)	0.857
	475	323	152	
HbA1c evolution				0.236
unchanged	22 (4.6)	12 (3.7)	10 (6.6)	
increased	89 (18.7)	57 (17.6)	32 (21.1)	
decreased	364 (76.6)	254 (78.6)	110 (72.4)	
	475	323	152	
Preeclampsia	197 (33.4)	134 (34.1)	63 (32.1)	0.703
	589	393	196	
Cesarean section	429 (74.0)	292 (75.1)	137 (71.7)	0.447
	580 (93.9)	389	191	
**Perinatal and neonatal outcomes**				
Pregnancy outcome				0.644
liveborn	562 (90.9)	376 (90.8)	186 (91.2)	
stillborn	17 (2.8)	13 (3.1)	4 (2.0)	
lost to follow-up	39 (6.3)	25 (6.0)	14 (6.9)	
	618	424	204	
Preterm birth	139 (24.0)	99 (25.5)	40 (21.1)	0.282
	578 (93.5)	388	190	
5 min Apgar < 7	23 (4.1)	18 (4.8)	5 (2.7)	0.342
	557	373	184	
Birth weight (g)	3278 (797)	3238 (829)	3358 (721)	0.089
	578	388	190	
Congenital anomaly	70 (12.6)	52 (14.0)	18 (9.8)	0.352
	556	372	184	
Macrosomia	87 (15.1)	59 (15.2)	28 (14.7)	0.981
	578	388	190	
Birth weight categories^b^				0.474
LGA	219 (38.4)	150 (39.1)	70 (37.0)	
AGA	322 (56.4)	217 (56.5)	106 (56.1)	
SGA	30 (5.3)	17 (4.5)	13 (6.9)	
	571	382	189	
Ventilatory disfunction	144 (26.2)	99 (27.0)	45 (24.6)	
	550	367	183	0.619
Hypoglycemia	117 (21.2)	82 (22.2)	35 (19.1)	0.467
	552	369	183	
Jaundice	185 (33.5)	125 (33.9)	60 (32.8)	0.873
	552	369	183	
Sepsis	103 (18.7)	71 (19.3)	32 (17.5)	0.681
	550	367	183	
NICU admission	234 (42.2)	168 (45.2)	66 (36.1)	0.051
	555	372	183	
Death^c^	32 (5.5)	25 (6.4)	7 (3.7)	0.245
	579	389	190	

Results presented as mean (standard deviation), n (%) or median (interquartile range)

*HCPA* Hospital de Clínicas de Porto Alegre, *HNSC* Hospital Nossa Senhora da Conceição, *HbA1c* glycated hemoglobin, *LGA* large for gestational age, *AGA* adequate for gestational age, *SGA* small for gestational age, *NICU* neonatal intensive care unit

Statistics: Student *t* test, the chi-square test (coupled with *Z* test for comparison of proportions, with Bonferroni correction when appropriate), and the Mann–Whitney *U* test

^a^According to the Institute of Medicine recommendation

^b^According to the World Health Organization chart

^c^Includes perinatal and neonatal death

**Fig. 2:**
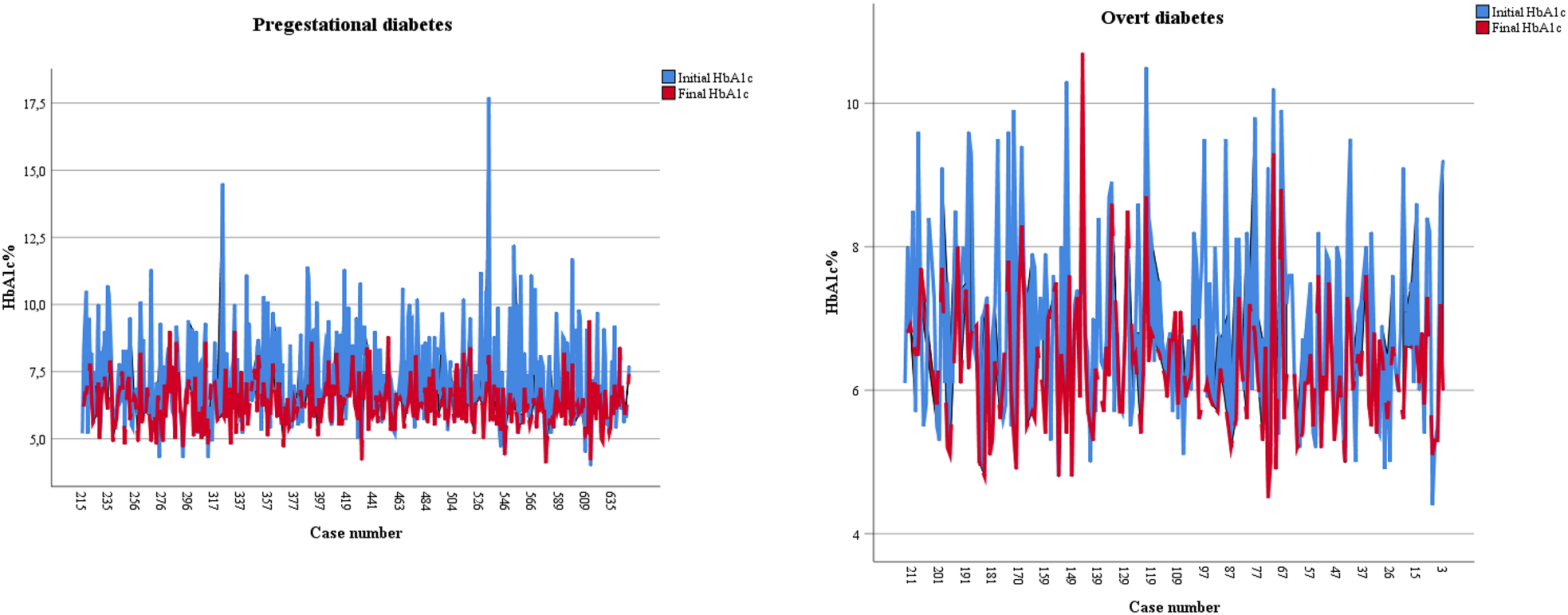
Evolution of glycated hemoglobin in pregnant women with pregestational or overt diabetes. *HbA1c* glycated hemoglobin

In Table 3, we present pregnancy outcomes according to the HbA1c measured at ≥ 28 weeks stratified by the cutoff value of 6.5%. HbA1c decreased from baseline values in most women (76.6%). An HbA1c ≥ 6.5% in the third trimester was associated with worse outcomes for mother and neonate (all outcomes, except death). In Fig. 2 we illustrate the temporal trend between the baseline and the third trimester HbA1c for each woman; in general, HbA1c decreased along pregnancy in both groups and most women (59.0%) reached values < 6.5% in the third trimester.

**Table 3:** Third trimester glycated hemoglobin and pregnancy outcomes

Outcome		HbA1c		p
	All	< 6.5%	≥ 6.5%	
	n = 475 (100)	n = 282 (59)	n = 193 (41)	
**Maternal outcomes**				
Specialized appointments	8.0 [4.0–11.0]	8.0 [5.0–11.0]	6.0 [3.5–11.0]	< 0.001
		282	193	
Insulin use	415 (87.7)	234 (83.6)	181 (93.8)	0.001
	473	280	193	
Hospitalization due to DM	269 (59.0)	134 (49.8)	135 (72.2)	< 0.001
	456	269	187	
Weight gain categories^b^				< 0.001
less than recommended	169 (37.3)	122 (45.4)^a^	47 (25.5)^a^	
as recommended	115 (25.4)	70 (26.0)^a^	45 (24.5)^a^	
more than recommended	169 (37.3)	77 (28.6)^a^	92 (50.0)^a^	
	453	269	184	
Initial HbA1c < 6.5%	154 (32.4)	131(46.5)	23 (11.9)	< 0.001
	475	282	193	
HbA1c variation				0.060
unchanged	22 (4.6)	13 (4.6)^a^	9 (4.7)^a^	
increased	89 (18.7)	43 (15.2)^a^	46 (23.8)^a^	
decreased	364 (76.6)	226 (80.1)^a^	138 (71.5)^a^	
	475	282	193	
Preeclampsia	143 (30.6)	83 (29.7)	60 (31.9)	0.692
	467	279	188	
Cesarean section	347 (75.8)	205 (75.6)	142 (75.9)	> 0.999
	458	271	187	
**Perinatal and neonatal outcomes**				
Pregnancy outcome				0.775
liveborn	446 (93.9)	264 (93.6)	182 (94.3)	
stillborn	11 (2.3)	6 (2.1)	5 (2.6)	
lost to follow-up	18 (3.8)	12 (4.3)	6 (3.1)	
	475	282	193	
Preterm birth	90 (19.7)	45 (16.7)	45 (24.1)	0.069
	456	269	187	
5 min Apgar < 7	17 (3.9)	9 (3.5)	8 (4.4)	0.808
	441	259	182	
Birth weight (g)	3363 (723)	3281 (716)	3481 (718)	0.003
		269	187	
Congenital anomaly	53 (11.9)	20 (11.0)	24 (13.3)	0.545
	445	264	181	
Macrosomia	75 (16.5)	34 (12.6)	41 (21.9)	0.012
	456	269	187	
Birth weight categories^c^				0.002
LGA	173 (38.1)	84 (31.5)^a^	89 (47.6)^a^	
AGA	261 (57.5)	168 (62.9)^a^	93 (49.7)^a^	
SGA	20 (4.4)	15 (5.6)^a^	5 (2.7)^a^	
	454	267	187	
Hypoglycemia	90 (20.4)	40 (15.4)	50 (27.6)	0.003
	441	260	181	
Jaundice	148 (33.6)	73 (28.1)	75 (41.4)	0.005
	441	260	181	
Sepsis	76 (17.3)	31 (12.0)	45 (25.0)	0.001
	439	259	180	
Ventilatory disfunction	101 (23.0)	46 (17.8)	55 (30.6)	0.003
	439	259	180	
NICU admission	169 (38.1)	80 (30.5)	89 (49.2)	< 0.001
	443	262	181	
Death^d^	20 (4.4)	13 (4.8)	7 (3.7)	0.750
	457	270	187	

Results presented as mean (standard deviation), n (%) or median (interquartile range)

*HbA1c* glycated hemoglobin, *DM* diabetes mellitus, *LGA* large for gestational age, *AGA* adequate for gestational age, *SGA* small for gestational age, *NICU* neonatal intensive care unit

Statistics: Student *t* test, the chi-square test (coupled with *Z* test for comparison of proportions, with Bonferroni correction when appropriate), and the Mann–Whitney *U* test

^a^Denote differences between subgroups

^b^According to the Institute of Medicine recommendation

^c^According to the World Health Organization chart

^d^Includes perinatal and neonatal death

Maternal and neonatal outcomes were similar in women with pregestational diabetes and overt diabetes; therefore, we calculated relative risks of main pregnancy outcomes, according to some known determinants, for the whole group (Table 4). Risk of presenting preeclampsia was associated with a higher pregestational BMI (and was not associated with the third trimester HbA1c); insulin use decreased risk by 35%. Gestational weight gain more than recommended increased risk of macrosomia by almost two times; and a high third trimester HbA1c, by 34.0%, while higher pregestational BMI had little impact, 4.0%. Regarding NICU admission, a higher third trimester HbA1c increased the risk by 22.0%; being SGA doubled the risk, while being LGA increased the risk by 46.0%. Perinatal/neonatal death was more than three times higher in SGA babies and a higher initial HbA1c increased this risk by 27.0%.

**Table 4:** Risk of maternal and neonatal outcomes in pregnant women with diabetes

Predictor	Maternal outcome		Fetal and perinatal outcomes					
	Preeclampsia	p	Macrosomia	p	Admission to NICU	P	Death^a^	p
	Total sample = 444		Total sample = 436		Total sample = 424		Total sample = 567	
Center (HNSC)	1.335 (0.993–1.795)	0.056	1.011 (0.686–1.489)	0.958	0.837 (0.660–1.062)	0.144	1.075 (0.456–2.534)	0.869
Age	1.009 (0.985–1.033)	0.482	0.989 (0.957–1.023)	0.534	0.980 (0.960–0.999)	0.049		
Diabetes duration	1.005 (0.971–1.040)	0.775	0.992 (0.951–1.036)	0.724	1.022 (0.996–1.048)	0.099	1.525 (0.584–3.983)	0.389
Pregestational BMI	1.039 (1.025–1.054)	< 0.001	1.040 (1.016–1.065)	0.001	0.999 (0.983–1.015)	0.923		
Recommended GWG								
less	0.930 (0.636–1.360)	0.708	0.499 (0.244–1.021)	0.057	1.086 (0.771–1.532)	0.636		
more	1.255 (0.866–1.820)	0.230	1.928 (1.146–3.244)	0.013	1.215 (0.882–1.673)	0.233		
Insulin use	0.653 (0.451–0.946)	0.024	1.670 (0.600–4.643)	0.326	0.757 (0.509–1.126)	0.170		
Hospitalization								
First HbA1c							1.271 (1.107–1.459)	0.001
3rd trimester HbA1c	1.033 (0.874–1.221)	0.703	1.338 (1.117–1.604)	0.002	1.221 (1.083–1.376)	0.001		
Preeclampsia					1.354 (1.064–1.723)	0.014		
SGA (WHO chart)					1.964 (1.199–3.216)	0.007	3.461 (1.207–9.925)	0.021
LGA (WHO chart)					1.463 (1.124–1.904)	0.005	1.096 (0.459–2.618)	0.836
Prenatal appointments							0.906 (0810–1.014)	0.086

Results presented as adjusted relative risk and 95% confidence interval using Poisson regression with robust estimates. *3rd* trimester HbA1c refers to any HbA1c measured with a gestational age ≥ 28 weeks

*NICU* neonatal intensive care unit, *BMI* body mass index, *GWG* gestational weight gain, *HbA1c* glycated hemoglobin, *SGA* small for gestational age, *LGA* large for gestational age, *WHO* World Health Organization,

^a^Includes perinatal and neonatal death

## Discussion

In this large cohort of pregnant women with diabetes, one third was unaware of having hyperglycemia, thereafter labelled as overt diabetes cases. Their pregnancy outcomes were very similar to those of women with an already known pre-pregnancy diabetes. Some factors were associated with worse outcomes: high pregestational BMI was associated with higher risk of preeclampsia and macrosomia, while more than recommended weight gain was only associated with macrosomia; use of insulin decreased the risk of preeclampsia. NICU admission was associated with maternal preeclampsia and higher third trimester HbA1c, as well as with either LGA or SGA babies. Perinatal mortality was associated with higher maternal HbA1c at booking and with being SGA.

Preconception care is a fundamental cornerstone to ensure healthier and safer pregnancy outcomes in women with diabetes [15]. A thorough approach should ideally include women´s care by a multidisciplinary team, focusing on education about the impact of diabetes on pregnancy outcomes, especially upon congenital anomalies and perinatal morbidity and mortality; teaching and supporting diabetes self-management skills; and warranting effective contraceptive methods until the best possible metabolic control is achieved. Before encouraging conception, chronic complications of diabetes have to be addressed and potentially harmful treatments, such as some anti-hyperglycemic and anti-hypertensive drugs, substituted by medications considered safer in pregnancy, along with folate supplementation. Nevertheless, when we face real life, many of these recommendations are not accomplished, due to several factors, including having diabetes not diagnosed before pregnancy and/or lack or misuse of contraceptive methods. We had no information on contraception in women of this cohort.

Undiagnosed diabetes is prevalent, affecting primarily individuals living in low and middle-income countries, where rates can reach almost 50.0% in adults aged 20–79 years [16]. In Brazil, the estimated proportion of undiagnosed diabetes in adults is 31.9% [16]; and diabetes rates in women of childbearing age vary from 1.0% in the 18–24 years age group to 3.9% in those aged up to 44 years [17]. Globally, hyperglycemia affects 16.7% of pregnancies; of these, 9.1% are cases of type 1 or type 2 diabetes first diagnosed in pregnancy [16]. Few studies report rates of diabetes first detected in pregnancy. In a Canadian study, 2.6% of women diagnosed with gestational diabetes presented type 2 diabetes during the first year postpartum and were labelled as having had previous overt diabetes; this represents less than 0.2% of the total study sample [18]. In a Brazilian cohort of pregnant women with hyperglycemia, ~ 21.0% of 224 women presented overt diabetes [19]. The rate of undiagnosed diabetes in our cohort reflects rates described for non-pregnant adults, despite many women already presented traditional risk factors: age ≥ 35 years (~ 42%), the cutoff point for age currently recommended for diabetes screening [3, 20]; history of gestational diabetes in previous pregnancies (~ 30.0%); and family history of diabetes (~ 61.0%) [3]. In two published papers, women considered as having overt diabetes displayed baseline characteristics quite similar to those with pregestational type 2 diabetes [18, 21], as here. In an Italian cohort, 66.7% of non-pregnant women with type 2 diabetes at childbearing age presented at least one high risk pre-conceptional feature, as obesity or hypertension [22]. In our cohort, if women with overt diabetes were screened before pregnancy with FPG or HbA1c due to their risk factors, they would have been diagnosed earlier, as 58.8% had at least one positive diagnostic criterion. Therefore, many women diagnosed with overt diabetes in our study are, most likely, women with diabetes antedating pregnancy that went unrecognized due to several factors, among them a low educational level and/or living in deprived settings. Social vulnerability was associated with worse control of diabetes in pregnancy in women with pregestational diabetes [23]; the same reasoning can be applied to cases of diabetes undiagnosed before pregnancy.

The comparison of baseline characteristics of women in our cohort to those of women with type 2 diabetes from other cohorts revealed similar maternal age, in the thirties [7, 8, 18, 21]. Obesity was almost as frequent (65.0% [7]; 51.6% [8]; and here, ~ 70.0%). In Brazil, obesity is present in 11.2% of women aged 18–24 years and in 25.7% of those aged 35–44 years, with higher rates in women with lower schooling [17]. Obesity has been increasing over the years [24], but in this cohort of pregnant women with type 2 diabetes, astonishing high rates (~ 70%) were seen during the study time span. Chronic hypertension was frequent in other cohorts − 12.0% [18]; 18.7% [21]; 10.3% [7]; and 16.4% [8], but here we saw the highest rate, ~ 23.0%. Women of childbearing age in Brazil have high rates of chronic hypertension, ~ 25% [17]. When we compared the Canadian women with overt diabetes [18] to the group of women with overt diabetes herein, rates of maternal age ≥ 26 years (88.2%) and previous gestational diabetes (30.4%) were similar; and previous hypertension rates were much lower in other cohorts [8, 21], highlighting a riskier profile of Brazilian women. Insulin resistance may play a pivotal role in the pathogenesis of obesity, type 2 diabetes and preeclampsia. Here, insulin treatment decreased the risk of preeclampsia in ~ 30.0%. As hyperglycemia may promote a pro-inflammatory environment, lowering glycemic levels with insulin treatment could have had a protective effect [25].

Importantly, the majority of women with overt diabetes was diagnosed in the first trimester, different from another study [21], but reached specialized prenatal care almost a month after their counterparts with pregestational diabetes, despite having similar rates of well-known risk factors. But even women with known pregestational diabetes arrived late, here. This is in contrast with results of a large study: women with pregestational diabetes arrived earlier, around the 9^th^ week; even though, only 22.0% of those women reported use of folate supplementation, a key feature of preconception care [7].

Other maternal factors were relevant. HbA1c values were very similar between groups, as well as the pattern of weight gain, with only ~ 26.0% of women gaining weight in the recommended range. HbA1c improved in most women, and in those with a baseline HbA1c ≥ 6.5%, more than half reached values < 6.5% in the third trimester. As expected, higher third trimester HbA1c was associated with worse pregnancy outcomes, mainly neonatal, reflecting the effects of sustained intrauterine hyperglycemia. These findings are in agreement with those described by others: a third trimester HbA1c ≥ 6.5% was associated with an almost fourfold increased risk of perinatal death [7].

Preterm birth rates were similar to those described in other studies [7, 8]. Comparing women with overt diabetes in the Canadian study [18] to those in our study, ~ 15.0% of women delivered before 37 gestational weeks vs. ~ 21.0%, respectively. Rates of cesarean section here (~ 75.0%) were the highest, compared to other samples; and 50% higher than the general rate of cesarean section in Brazil (~ 56%) [26]. Determinants of cesarean section are complex in our country, therefore limiting comparisons.

Neonatal outcomes were quite different from those of other cohorts. Congenital malformations were at least two times as frequent here (12.6%), compared either to women with pregestational type 2 diabetes (4.0% [7]) or to women with overt diabetes (5.6% [7]; 1.1% [21]). Comparison among series may be biased by different methods of investigation and classification. The high frequency may also point to a lack of preconception care in our series, since women began pregnancy with a mean value HbA1c of 7.2%, compared to 6.9% in another study [7]; women with pre-pregnancy diabetes displayed significantly higher values of HbA1c, similar to what was already described [21].

Macrosomia occurred at rates similar to another study, ~ 15.0% [8]. Rates of SGA varied widely [7, 21], and, among women with overt diabetes, rates were 6.9% here, compared to 9.5% [18] and 11.0% [21] in other studies. Around 23% of the SGA babies in our cohort had some congenital anomaly or chromosomal disorder. LGA babies were common across series, more than 20.0% [7, 8, 18, 21]; and in women with undiagnosed diabetes, rates were ~ 22.0% [18, 21], compared to 37.0% here. Not surprisingly, delivery of LGA babies was ~ 38.0% and of macrosomic babies, ~ 15%: pregestational obesity was present in ~ 70.0% of women, and ~ 37.0% had more than recommended weight gain during pregnancy. Both situations, especially preconception obesity, are known risk factors for delivering heavier babies [27]. Comparisons of birth weight categories must be interpreted with caution, because rates depend on the reference chart used.

Neonatal hypoglycemia was reported in 26.4% in women with undiagnosed diabetes [18], compared to 19.1% here. The lower rate of hypoglycemia here could be due to the use of the same management recommended for pregestational diabetes during pregnancy, labor and delivery.

In our study, 42.0% of the babies were admitted to the NICU, compared to lower rates in other studies [7, 8]. In women with overt diabetes, rate was 24.3% [18], compared to 36.3% here. NICU admission was associated with maternal preeclampsia, with delivery of SGA or LGA babies, and also with a higher maternal third trimester HbA1c, highlighting the relevance of an adequate glycemic control throughout pregnancy.

Perinatal and neonatal death were associated with higher values of HbA1c at booking. An adverse intrauterine hyperglycemic milieu at conception and organogenesis period leads to a higher risk of congenital malformations, SGA babies, and perinatal death [15]. Death of SGA babies was more frequent here; congenital anomalies and fetal growth restriction might be the imputable factors [28].

In these women, several adverse pregnancy outcomes could have been avoided if they had been prepared to conceive. Preconception care was associated with substantial reduction in rates of congenital malformations and admission to NICU, independent of early prenatal care [15]. Adequate metabolic control, expressed by lower HbA1c values in early pregnancy, was also more frequent in women receiving preconception care. Nevertheless, even women with known diagnosis of diabetes arrived late to prenatal care and with higher than recommended HbA1c values. In most women, HbA1c decreased along pregnancy, reaching recommended values at delivery in 59.0% of women; despite this, at least one clinically relevant adverse outcome occurred in 41.9% of the neonates. A long road towards prioritizing the women’s health at childbearing age, especially during preconception and early pregnancy, is still ahead. And hyperglycemia undoubtedly contributes to an unhealthy intrauterine milieu.

To our knowledge, this is the first manuscript comparing, head-to-head, the pregnancy outcomes of women with known pregestational diabetes to those of women with overt diabetes, being this the main strength of the study. Other studies looked at these women´s pregnancies; in one, overt diabetes was diagnosed retrospectively [18]; in another, no direct comparisons were performed between the two groups [21]; most studies compared pregnancy outcomes of women with overt diabetes with those of women with gestational diabetes [29]. Other strength is the large number of women evaluated in the two major regional high-risk maternities. We disclosed a high frequency of overt diabetes, denoting women with unknown hyperglycemia at conception despite displaying several classic risk factors. We should promote actions for prompt diagnosis of these women before pregnancy, since they are at the same risk level of adverse pregnancy outcomes as women with known diabetes; and for sensitizing the primary care health team to educate women of childbearing age about the benefits of preconception care, especially in the presence of obesity or hyperglycemia.

Main limitations were due to retrospective and secondary data collection, despite all women had been prospectively followed by medical teams in the two maternities. Some risk factors were assumed as negative when not recorded in the hospital charts, thus potentially underestimating their impact. We used clinical features to label type 2 diabetes, despite the current trend to include genetic and metabolic testing to refine diabetes subtypes classification [30]. Therefore, women with MODY could had been labelled as having type 2 diabetes, since genetic tests were not available at the two hospitals. Of note, MODY accounts for only ~ 1.0% of the cases of diabetes and pregnancy [31]; therefore, the impact on results would be very small if we had to exclude such cases. Nearly 25% of women with pregestational diabetes used insulin, alone or in combination with oral medications, raising questions about whether some of them might be cases of LADA. Nevertheless, clinical features matched those of type 2 diabetes and we had excluded those suspected of having LADA. We also accepted diagnosis of preeclampsia, neonatal hypoglycemia and the criteria for admission to NICU as defined by the medical teams. Slight differences in diabetes management and outcomes definitions between the centers did not translate into different outcomes. Finally, although many women with overt diabetes might return to a normal glycemic condition after pregnancy [29], we could not fully reclassify their glycemic status after delivery; however, low return rates to retest for diabetes were previously reported [21].

## Conclusion

Overt diabetes was diagnosed in one third of this cohort of pregnant women with hyperglycemia. Their pregnancy outcomes were similar to those of women with pregestational diabetes and were mostly related to maternal demographic characteristics and metabolic control. A call to action should be made to identify women of childbearing age at risk for pre-pregnancy diabetes; to detect hyperglycemia before conception; and to implement timely preconception care to all women with diabetes.

## Supplementary Information

**Additional file 1: Table S1.** Baseline maternal characteristics and pregnancy outcomes by center.

## Data Availability

The datasets used and/or analyzed during the current study are available from the corresponding author on reasonable request. Inquiries can be directed to the corresponding author (areichelt@hcpa.edu.br) or to Vania Hirakata (vhirakata@hcpa.edu.br).

## Abbreviations

AGA: Adequate for gestational age
BMI: Body mass index
CH: Chronic hypertension
CI: Confidence interval
DM: Diabetes mellitus
FPG: Fasting plasma glucose
GWG: Gestational weight gain
HbA1c: Glycated hemoglobin
HCPA: Hospital de Clínicas de Porto Alegre
HNSC: Hospital Nossa Senhora da Conceição
IQR: Interquartile range
LADA: Latent autoimmune diabetes of adulthood
LGA: Large for gestational age
MODY: Maturity-onset diabetes of young
NICU: Neonatal intensive care unit
OGTT: Oral glucose tolerance test
RR: Relative risk
SD: Standard deviation
SGA: Small for gestational age
STROBE: Strengthening the Reporting of Observational Studies in Epidemiology
WHO: World Health Organization

## References

[1] Yu L, Zeng XL, Cheng ML, Yang GZ, Wang B, Xiao ZW (2017). Quantitative assessment of the effect of pre-gestational diabetes and risk of adverse maternal, perinatal and neonatal outcomes. Oncotarget.

[2] Simmons D (2021). Paradigm shifts in the management of diabetes in pregnancy: the importance of type 2 diabetes and early hyperglycemia in pregnancy: the 2020 Norbert Freinkel award lecture. Diabetes Care.

[3] Classification and Diagnosis of Diabetes (2022). Standards of medical care in diabetes-2022. Diabetes Care.

[4] Zajdenverg L, Façanha CFS, Dualib PM, Golbert A, Moisés ECD, de Calderon IMP, et al. Rastreamento e diagnóstico da hiperglicemia na gestação: Sociedade Brasileira de Diabetes. 2022 (updated 2021-12-02). https://diretriz.diabetes.org.br/rastreamento-e-diagnostico-da-hiperglicemia-na-gestacao/. Accessed 9 Oct 2022

[5] World Health Organization (2014). Diagnostic criteria and classification of hyperglycaemia first detected in pregnancy: a World Health Organization Guideline. Diabetes Res Clin Pract.

[6] Balsells M, García-Patterson A, Gich I, Corcoy R (2009). Maternal and fetal outcome in women with type 2 versus type 1 diabetes mellitus: a systematic review and metaanalysis. J Clin Endocrinol Metab.

[7] Murphy HR, Howgate C, O'Keefe J, Myers J, Morgan M, Coleman MA (2021). Characteristics and outcomes of pregnant women with type 1 or type 2 diabetes: a 5-year national population-based cohort study. Lancet Diabetes Endocrinol.

[8] Newman C, Egan A, Ahern T, Al-Kiyumi M, Balan G, Brassill M (2021). Diabetes care and pregnancy outcomes for women with pregestational diabetes in Ireland. Diabetes Res Clin Pract.

[9] Hospital de Clínicas de Porto Alegre: Hospital de Clínicas de Porto Alegre. 2021. https://www.hcpa.edu.br/institucional/institucional-apresentacao/institucional-apresentacao-principais-numeros. Accessed 9 Oct 2022.

[10] Hospital Nossa Senhora da Conceição: Grupo Hospital Conceição; 2021. https://www.ghc.com.br/default.asp?idMenu=acessoinformacao&idSubMenu=12. Accessed 9 Oct 2022.

[11] Rodacki M, Teles M, Gabbay M, Montenegro R, Bertoluci M. Classificação do diabetes: sociedade Brasileira de Diabetes. 2022. https://diretriz.diabetes.org.br/classificacao-do-diabetes/. Accessed 9 Oct 2022.

[12] Rasmussen KM, Yaktine AL, Institute of Medicine and National Research Council Committee to Reexamine IOMPWG (2009). The national academies collection reports funded by national institutes of health. Weight gain during pregnancy: reexamining the guidelines.

[13] Villar J, Cheikh Ismail L, Victora CG, Ohuma EO, Bertino E, Altman DG (2014). International standards for newborn weight, length, and head circumference by gestational age and sex: the Newborn Cross-Sectional Study of the INTERGROWTH-21st Project. Lancet.

[14] von Elm E, Altman DG, Egger M, Pocock SJ, Gøtzsche PC, Vandenbroucke JP (2007). The Strengthening the Reporting of Observational Studies in Epidemiology (STROBE) statement: guidelines for reporting observational studies. Lancet.

[15] Wahabi HA, Fayed A, Esmaeil S, Elmorshedy H, Titi MA, Amer YS (2020). Systematic review and meta-analysis of the effectiveness of pre-pregnancy care for women with diabetes for improving maternal and perinatal outcomes. PLoS ONE.

[16] IDF Diabetes Atlas Brussels, Belgium. 10th ed; 2021. https://www.diabetesatlas.org. Accessed 9 Oct 2022.

[17] Brasil, Ministério da Saúde. Vigitel Brasil 2020: vigilância de fatores de risco e proteção para doenças crônicas por inquérito telefônico: estimativas sobre frequência e distribuição sociodemográfica de fatores de risco e proteção para doenças crônicas nas capitais dos 26 estados brasileiros e no Distrito Federal em 2020/Ministério da Saúde, Secretaria de Vigilância em Saúde, Departamento de Análise em Saúde e Vigilância de Doenças Não Transmissíveis—Saúde Md, editor. Brasília: Ministério da Saúde; 2021. p. 124

[18] Lee D, Booth GL, Ray JG, Ling V, Feig DS (2020). Undiagnosed type 2 diabetes during pregnancy is associated with increased perinatal mortality: a large population-based cohort study in Ontario. Canada Diabet Med.

[19] Sampaio Y, Porto L, Lauand T, Marcon L, Pedrosa H (2021). Gestational diabetes and overt diabetes first diagnosed in pregnancy: characteristics, therapeutic approach and perinatal outcomes in a public healthcare referral center in Brazil. Arch Endocrinol Metab.

[20] Davidson KW, Barry MJ, Mangione CM, Cabana M, Caughey AB, Davis EM (2021). Screening for prediabetes and Type 2 diabetes: US preventive services task force recommendation statement. JAMA.

[21] Immanuel J, Eagleton C, Baker J, Simmons D (2021). Pregnancy outcomes among multi-ethnic women with different degrees of hyperglycaemia during pregnancy in an urban New Zealand population and their association with postnatal HbA1c uptake. Aust N Z J Obstet Gynaecol.

[22] Scavini M, Rossi MC, Scardapane M, Nicolucci A, Manicardi V, Russo G (2018). Portrait of women with type 1 or type 2 diabetes of childbearing age attending diabetes clinics in Italy: the AMD-Annals initiative. Acta Diabetol.

[23] Venkatesh KK, Germann K, Joseph J, Kiefer M, Buschur E, Thung S (2022). Association between social vulnerability and achieving glycemic control among pregnant individuals with pregestational diabetes. Obstet Gynecol.

[24] (NCD-RisC) NRFC (2016). Trends in adult body-mass index in 200 countries from 1975 to 2014: a pooled analysis of 1698 population-based measurement studies with 19·2 million participants. Lancet.

[25] van Niekerk G, Christowitz C, Engelbrecht AM (2021). Insulin-mediated immune dysfunction in the development of preeclampsia. J Mol Med.

[26] Rudey EL, Leal MDC, Rego G (2020). Cesarean section rates in Brazil: trend analysis using the Robson classification system. Medicine.

[27] Creanga AA, Catalano PM, Bateman BT (2022). Obesity in pregnancy. N Engl J Med.

[28] Damhuis SE, Ganzevoort W, Gordijn SJ (2021). Abnormal fetal growth: small for gestational age, fetal growth restriction, large for gestational age: definitions and epidemiology. Obstet Gynecol Clin North Am.

[29] Goyal A, Gupta Y, Tandon N (2022). Overt diabetes in pregnancy. Diabetes Ther.

[30] Deutsch AJ, Ahlqvist E, Udler MS (2022). Phenotypic and genetic classification of diabetes. Diabetologia.

[31] Egan AM, Simmons D (2019). Lessons learned from lifestyle prevention trials in gestational diabetes mellitus. Diabet Med.

