# Supplementary material for: Overt diabetes imposes a comparable burden on outcomes as pregestational diabetes: a cohort study
Source: Diabetol Metab Syndr. 2022 Nov 23;14:177. doi: 10.1186/s13098-022-00939-1 (PMC9685976; doi:10.1186/s13098-022-00939-1)
Supplement: Supplementary file 1 — Additional file 1: Table S1. Baseline maternal characteristics and pregnancy outcomes by center. [file 13098_2022_939_MOESM1_ESM.docx]

Suppl Table 1. Baseline maternal characteristics and pregnancy outcomes by center

| **Characteristic** |  | **Center** | |  |  |
| --- | --- | --- | --- | --- | --- |
|  | **All** | **HCPA** | **HNSC** | **p** |  |
|  | **618 (100)** | **284 (46)** | **334 (54)** |  |  |
| **Maternal baseline characteristics** | | | | |  |
| Age | 32.8 (5.9) | 32.9 (5.8) | 32.7 (6.0) | 0.74 |  |
| White skin color | 433 (70.1) | 218 (76.8) | 215 (64.4) | <0.001 |  |
| Years in school ≤ 11 years | 588 (95.1) | 268 (94.4) | 320 (95.8) | 0.52 |  |
| Smoking | 52 (8.4) | 39 (13.7) | 13 (3.9) | <0.001 |  |
| Chronic hypertension | 143 (23.1) | 62 (21.8) | 81 (24.3) | 0.54 |  |
| Diabetes complication | 37 (6.0) | 21 (7.4) | 16 (4.8) | 0.23 |  |
| Treatment before pregnancy |  |  |  | <0.001 |  |
| diet | 23 (3.8) | 6 (2.1)^a^ | 17 (5.2)^b^ |  |  |
| oral medication | 248 (40.5) | 99 (34.9)^a^ | 149 (45.3)^b^ |  |  |
| insulin | 41 (6.7) | 30 (10.6)^a^ | 11 (3.3)^b^ |  |  |
| all | 63 (10.3) | 40 (14.1)^a^ | 23 (7.0)^b^ |  |  |
| none | 238 (38.8) | 109 (38.4)^a^ | 129 (39.2)^a^ |  |  |
|  | 613 (99.2) | 284 | 329 |  |  |
| Family history of diabetes | 416 (67.3) | 168 (59.2) | 248 (74.3) | <0.001 |  |
| Family history of CH | 309 (50.0) | 85 (29.9) | 224 (67.1) | <0.001 |  |
| First pregnancy | 114 (18.4) | 49 (17.3) | 65 (19.5) | 0.55 |  |
| Previous GDM | 195 (31.6) | 88 (31.0) | 107 (32.0) | 0.85 |  |
| Previous macrosomia | 124 (20.1) | 57 (20.1) | 67 (20.1) | > 0.99 |  |
| BMI categories |  |  |  | 0.003 |  |
| normal | 55 (9.2) | 14 (5.3)^a^ | 41 (12.4)^b^ |  |  |
| overweight | 117 (19.6) | 62 (23.4)^a^ | 55 (16.6)^b^ |  |  |
| obesity | 424 (71.1) | 189 (71.3)^a^ | 235 (71.0)^a^ |  |  |
|  | 596 | 265 | 331 |  |  |
| Gestational age at booking | 19.6 [14.0-27.4] | 18.1 [12.6-26.0] | 21.0 [15.3-28.8] | <0.001 |  |
| Trimester of enrollment |  |  |  | 0.001 |  |
| < 13 weeks | 123 (19.9) | 71 (25.0)^a^ | 52 (15.6)^b^ |  |  |
| ≥ 13-23 weeks | 275 (44.5) | 130 (45.8)^a^ | 145 (43.4)^a^ |  |  |
| ≥ 24 weeks | 220 (35.6) | 83 (29.2)^a^ | 137 (41.0)^b^ |  |  |
|  | 618 | 284 | 334 |  |  |
| HbA_1c_ at booking | 7.2 (1.5) | 7.3 (1.5) | 7.2 (1.6) | 0.85 |  |
|  | 612 | 278 | 334 |  |  |
| **Maternal outcomes** | | | | |  |
| GWG categories* |  |  |  | 0.96 |  |
| less than recommended | 211 (37.5) | 92 (37.2) | 119 (37.7) |  |  |
| as recommended | 146 (25.9) | 63 (25.5) | 83 (26.3) |  |  |
| more than recommended | 206 (36.6) | 92 (37.2) | 114 (36.1) |  |  |
|  | 563 | 247 | 316 |  |  |
| HbA_1c_ at ≥ 28 weeks | 6.3 (0.9) | 6.3 (0.9) | 6.3 (0.9) | > 0.99 |  |
|  | 475 | 221 | 254 |  |  |
| Diabetes hospitalization | 344 (58.7) | 142 (55.0) | 202 (61.6) | 0.13 |  |
|  | 586 | 258 | 328 |  |  |
| Pre-eclampsia | 197 (33.1) | 69 (25.7) | 128 (39.3) | 0.001 |  |
|  | 595 | 269 | 326 |  |  |
| Insulin | 511 (84.3) | 244 (88.1) | 267 (81.2) | 0.026 |  |
|  | 606 | 277 | 329 |  |  |
| Cesarean section | 429 (74.0) | 187 (70.8) | 242 (76.6) | 0.14 |  |
|  | 580 | 264 | 316 |  |  |
| **Perinatal and neonatal outcomes** | | | | |  |
| Perinatal outcome |  |  |  | 0.78 |  |
| liveborn | 562 (90.9) | 256 (90.1) | 306 (91.6) |  |  |
| stillborn | 17 (2.8) | 8 (2.8) | 9 (2.7) |  |  |
| lost to follow-up | 39 (6.3) | 20 (7.0) | 19 (5.7) |  |  |
|  | 618 | 284 | 334 |  |  |
| Birth weight | 3278 (797) | 3285 (830) | 3271 (769) | 0.83 |  |
|  | 578 | 264 | 314 |  |  |
| Preterm birth | 139 (24.0) | 72 (27.3) | 67 (21.3) | 0.12 |  |
|  | 578 | 264 | 314 |  |  |
| Birthweight category ** |  |  |  | 0.079 |  |
| SGA | 30 (5.3) | 10 (3.9) | 20 (6.4) |  |  |
| AGA | 322 (56.4) | 138 (53.3) | 184 (59.0) |  |  |
| LGA | 219 (38.4) | 111 (42.9) | 108 (34.6) |  |  |
|  | 571 | 259 | 312 |  |  |
| Macrosomia | 87 (15.1) | 40 (15.2) | 47 (15.0) | > 0.99 |  |
|  | 578 | 264 | 314 |  |  |
| Hypoglycemia | 117 (21.2) | 52 (20.6) | 65 (21.7) | 0.85 |  |
|  | 552 | 252 | 300 |  |  |
| NICU admission | 234 (42.2) | 109 (43.1) | 125 (41.4) | 0.75 |  |
|  | 555 | 253 | 302 |  |  |
| Death**** | 32 (5.5) | 14 (5.3) | 18 (5.7) | 0.97 |  |
|  | 579 | 264 | 315 |  |  |

* according to the Institute of Medicine recommendation

** according to the World Health Organization chart

*** includes perinatal and neonatal death

HCPA: Hospital de Clínicas de Porto Alegre; HNSC: Hospital Nossa Senhora da Conceição; CH: chronic hypertension; BMI: body mass index; HbA_1c_: glycated hemoglobin; GWG: gestational weight gain; SGA: small for gestational age; AGA: adequate for gestational age; LGA: large for gestational age; NICU: neonatal intensive care unit

Results presented as mean (standard deviation), n (%) or median [interquartile range]
